# Disease of the Tarsal Bones—Chopart’s Operation

**Published:** 1842-08-13

**Authors:** 


*Disease of the Tarsal Bones—Chopart's Operation.*—H. S., a pale, unhealthy-looking lad, of sallow complexion and slight conformation, was admitted into the University College Hospital April 7, under the care of Mr. Liston. Just before Christmas, in jumping from a height, he received a very severe sprain of the foot. This obliged him to lay up, and was followed by considerable inflammation of the foot, and the formation, after many weeks, of an abscess on the outer side of the foot, over the cuboid bone.—About three weeks before his admission this abscess was opened, and discharged a considerable quantity of pus. An unhealthy-looking ulcer now exists in this place, surrounded with blue-looking integument, filled with pale granulations, and attended with much ichorous discharge. The grating of carious bone can be felt by the probe, passed through a sinus opening into the ulcer. He was immediately placed in bed, and the leg and foot kept quite at rest, the ulcer being dressed with simple water-dressing. He was placed on full diet, and ordered to take the iodide of iron. No change taking place, it was next attempted to expose the diseased bone, by destroying the granulations of the ulcer with potassa fusa. It was soon, however, discovered that the disease extended through the articulation, between the calcaneum and the cuboid and scaphoid bones, so that a probe passed readily from the ulcer, through the articulation, to the inside of the foot. His health seemed to suffer considerably, and after a month's residence in the hospital, he was sent out for change of air.

On the 12th of April, five days after leaving the hospital, he returned again, suffering severe pain in the diseased foot, so much as to entirely prevent sleep: he had become very thin, and exhibited all the symptoms of hectic fever. Under these circumstances Mr. Liston proposed an operation, to which the boy consented.

13. To-day Mr. Liston removed the whole of the foot, with part of the astragalus and os calcis, after Chopart's method. The articulating surfaces of the calcis, cuboid, and scaphoid, were found completely deprived of their cartilage by ulceration, and the two latter bones evidently diseased. A small portion of the os calcis, also diseased, was removed by the cutting pliers. Two bleeding vessels were secured by ligature; and in the evening, bleeding having entirely ceased, the flap was placed in position, and retained by three points of suture and isinglass plaster. He was placed in bed, and the leg raised on the double-inclined plane (MacIntyre's apparatus).

14. Has passed a good night; feels better, and more free from pain than he has for some weeks before; looks cheerful. To have one grain of the iodide of iron in an ounce of infusion of orange-peel, three times a-day.

15. Feels in every respect much better, excepting that he is a little feverish. Bowels have not been opened for two days. To have half an ounce of castor-oil directly.

16. Has slept but little during the night, having been in much pain from some displacement of the apparatus in which the leg rests. This was re-adjusted to his comfort. Bowels have been freely opened; pulse 120; tongue clean and moist.

24. From the last report everything has gone on well, the greater part of the flap having united very firmly. At two points there is some degree of suppuration going on deeply, and communicating with the surface by two small sinuses. The ununited portion of flap is dressed daily with solution of sulphate of zinc and lint, and oil-silk, and is cicatrising rapidly. His health is greatly improved.

May 2. The treatment continues the same, with the addition of a roller, and the case is going on favourably.

23. After the last report, two small abscesses formed near the heel, which were opened, and soon healed, and he was discharged at this date. He has returned once since, and the portion of the foot left seems likely to be very serviceable. The operation does not seem at all likely to be followed by that displacement of the foot backwards which sometimes follows it, and which by some is considered a serious objection to it.—*London Lancet, June* 25, 1842.

